# Activation of the Epstein–Barr virus lytic cycle by the latex of the plant *Euphorbia tirucalli*

**DOI:** 10.1038/sj.bjc.6600929

**Published:** 2003-05-13

**Authors:** A MacNeil, O P Sumba, M L Lutzke, A Moormann, R Rochford

**Affiliations:** 1 Department of Epidemiology, School of Public Health, University of Michigan, Ann Arbor, MI, USA; 2 Kenyan Medical Research Institute, Kisian, Kenya; 3 Division of Geographic Medicine, Case Western Reserve University, Cleveland, OH, USA

**Keywords:** Epstein–Barr virus, Burkitt's lymphoma, *E. tirucalli*, viral gene expression

## Abstract

Exposure to the plant *Euphorbia tirucalli* has been proposed to be a cofactor in the genesis of endemic Burkitt's lymphoma (eBL). The purpose of this study was to examine the effects of unpurified *E. tirucalli* latex on Epstein–Barr virus (EBV) gene expression. A Burkitt lymphoma cell line was treated with varying dilutions of the latex and the effects on EBV gene expression were measured. We observed that the latex was capable of reactivating the EBV lytic cycle in a dose-dependent manner and at dilutions as low as 10^−6^. Simultaneous treatment of cells with *E. tirucalli* latex and the protein kinase C inhibitor 1-(5-isoquinolinesulphonyl)-2-methylpiperazine dihydrochloride blocked lytic cycle activation. These data suggest that environmental exposure to the latex of *E. tirucalli* could directly activate the EBV lytic cycle and provide further evidence of a role for *E. tirucalli* in the aetiology of eBL.

Endemic Burkitt's lymphoma (eBL) is a monoclonal B-lymphocytic malignancy and is the most common childhood cancer in sub-Saharan Africa (de-The, 1985; Crawford, 2001). Infection with Epstein–Barr virus (EBV) and the occurrence of holoendemic malaria are two clearly defined agents that are associated with the development of eBL (de-The, 1985; Morrow, 1985). However, the plant *Euphorbia tirucalli*, a member of the Euphorbiaceae family, has also been proposed to be a cofactor in the development of eBL (Osato *et al*, 1987; van den Bosch *et al*, 1993).

Methanol extracts of the stalks, leaves and roots of *E. tirucalli* enhance EBV-mediated cell transformation (Mizuno *et al*, 1983), modulate EBV-specific T cell activity, and induce chromosomal translocations in B cells (Imai *et al*, 1994), giving biological plausibility to a mechanism that links exposure to *E. tirucalli* with eBL. Furthermore, the geographical distribution of *E. tirucalli* is consistent with the incidence of eBL in Africa. For example, *E. tirucalli* is abundant in areas of Kenya and Tanzania that have high rates of eBL – most notably in the Lake Victoria Basin of Kenya– but is not seen in areas of Kenya where eBL is uncommon (Mizuno *et al*, 1983; Osato *et al*, 1987). In addition, children with eBL in Malawi had a significantly greater incidence of *E. tirucalli* growing around the homes relative to controls (van den Bosch *et al*, 1993).

*E. tirucalli* plants have a milky latex that is commonly used as glue and is played with by children in Western Kenya, an area with a high rate of eBL (MacNeil, Sumba, Rochford, unpublished observations), suggesting that exposure to *E. tirucalli* could occur through direct contact with the latex. However, the biological activity of the unpurified latex is unknown. *E. tirucalli* extracts contain 4-deoxyphorbol ester (Osato *et al*, 1987), a compound similar to 12-*O*-tetradecanoylphorbol-13-acetate (TPA), a known inducer of the EBV lytic cycle in latently-infected B cells (zur Hausen *et al*, 1978; Miller, 1990; Baumann *et al*, 1998). Evidence of elevated antibody titres against the EBV viral lytic cycle capsid antigen preceding development of BL (de-The, 1985) suggests that viral reactivation is associated with increased risk for lymphoma development in the context of malaria-induced immunosuppression.

In this study, we examined whether unpurified *E. tirucalli* latex could reactivate the viral lytic cycle in a manner analogous to TPA. We found that very dilute concentrations of unpurified *E. tirucalli* latex induced viral reactivation, providing further support for the hypothesis that exposure to *E. tirucalli* could be a cofactor in the genesis of eBL.

## MATERIALS AND METHODS

### Cell culture

The Jijoye cell line was obtained from the American Type Culture Collection (Rockville, MD, USA) (ATCC #CCL87). Peripheral blood lymphocytes (PBL) were isolated as previously described (Rochford and Mosier, 1995). Cells were maintained in complete medium (CM) which contained RPMI 1640 medium (Gibco-BRL, Bethesda, MD, USA) supplemented with 10% foetal bovine serum (Gemini BioProducts, Woodland, CA, USA), 2 mM L-glutamine and penicillin–streptomycin (Gibco-BRL).

### *E. tirucalli* latex

*E. tirucalli* plants were grown in the laboratory. The latex was extracted aseptically from *E. tirucalli* and diluted in CM. Fresh latex was extracted for each experiment.

### RNA extraction and ribonuclease protection assay (RPA)

RNA was extracted from cells (Chomczynski and Sacchi, 1987) and RPAs were performed exactly as described (Rochford *et al*, 1997). The riboprobe templates for BZLF1, gp350 and rpL32 have been previously described (Rochford *et al*, 1997). A riboprobe template was made for EAD as described (nt: 79978-80160; Genbank Accession number V01555) (Rochford *et al*, 1997). Quantitation of the RPA was done using a Storm PhosphorImager and ImageQuant software (Molecular Dynamics, Sunnyvale, CA, USA). Volume measurements with rectangular objects were used to generate PhosphorImager (PI) counts for each protected band; these were presented as a percentage of the internal housekeeping signal (i.e. rpL32).

### Cell treatment with *E. tirucalli* latex

For all experiments, Jijoye cells in the logarithmic phase of growth were seeded at a concentration of 7.5 × 10^5^ cells ml^−1^. Cells were treated with CM, CM plus varying dilutions of *E. tirucalli* latex, with *E. tirucalli* latex and protein kinase C inhibitor 1-(5-isoquinolinesulphonyl)-2-methylpiperazine (PKC-I) (Sigma Chemical Co., St Louis, MO, USA), or with TPA (Sigma Chemical).

### Immunofluorescence (IF) staining and flow cytofluorimetric (FCF) analysis

The primary antibody was a mouse anti-EBV MA-gp350/220 monoclonal antibody (clone 2L10, Chemicon International, Temecula, CA, USA). Fluorescein isothiocyanate-conjugated polyclonal goat-anti-mouse IgG (Sigma Chemical Co.) was used as the second label. Jijoye cells, treated with CM, CM supplemented with latex diluted 10^−3^ or with 10 ng ml^−1^ TPA, were analysed for gp350 expression using a FACSCalibur (BD Biosciences, San Jose, CA, USA).

## RESULTS

### *E. tirucalli* latex induces expression of EBV lytic cycle genes

**Figure 1:**
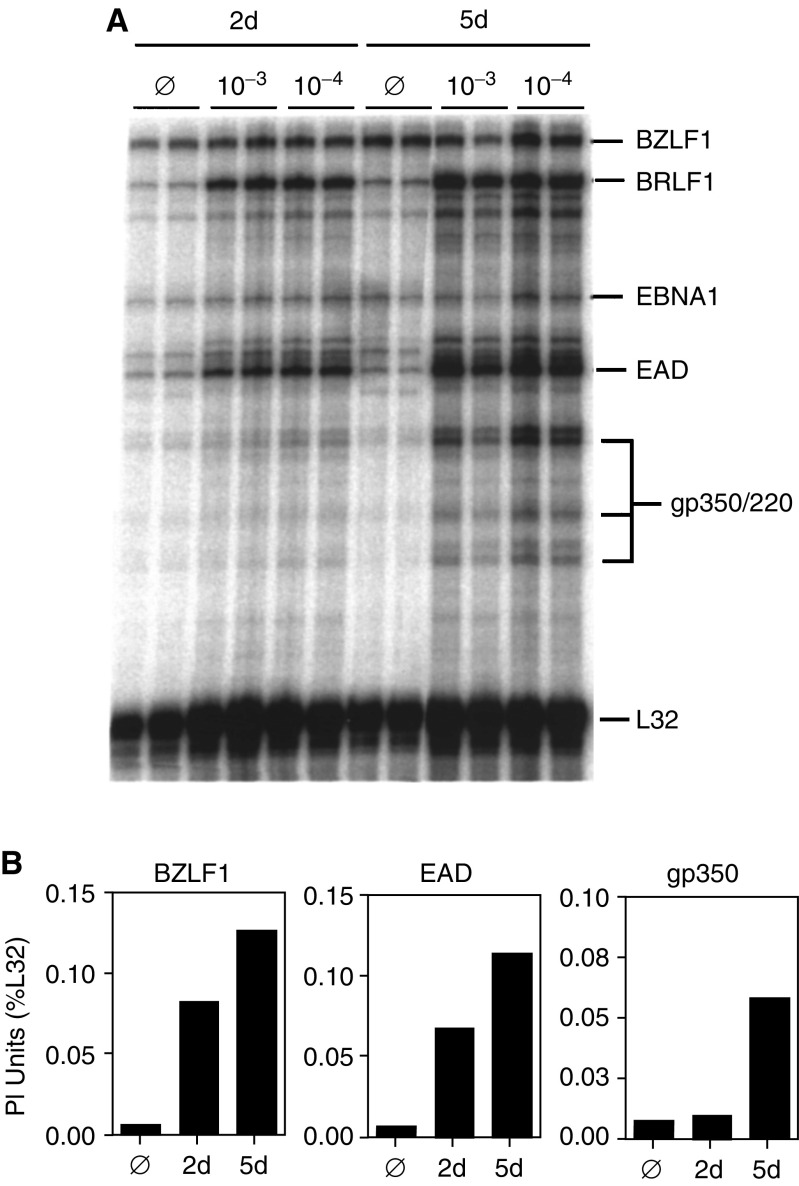
Effect of *E. tirucalli* latex on EBV lytic gene expression. (**A**) A multiprobe RNase protection assay was performed to detect EBV latent (EBNA-1) and lytic (BZLF1, BRLF1, EAD and gp350/220) mRNA. Jijoye cells were left untreated (∅) or treated with a 10^−3^ or 10^−4^ dilution of *E. tirucalli* latex for 2 (2 d) or 5 days (5 d). RNA was extracted, and RNA from 3 × 10^5^ cells was analysed in each track. Samples were done in duplicate. (**B**) The PI counts for each protected probe fragment were obtained, and the data are presented as a percentage of the internal housekeeping (i.e. L32) signal present in each lane. The average values from three experiments are presented.

To determine if the unpurified *E. tirucalli* latex removed directly from the plant could induce expression of EBV lytic cycle genes, the Jijoye BL cell line was treated with or without *E. tirucalli* latex diluted to 10^−3^ or 10^−4^ in CM. At 2 and 5 days post-treatment, RNA was extracted and an RPA using an EBV-specific riboprobe set was performed to assess the level of expression of EBV lytic cycle genes. Figure 1A

shows a representative RPA from three separate experiments and the quantitation is shown in Figure 1B. We quantitated expression of BZLF1, EAD and gp350 genes, which are in the immediate-early, early and late kinetic classes of lytic cycle expression, respectively. Untreated Jijoye cells had a very low level of lytic cycle gene expression consistent with previous studies (Miller, 1990). Following treatment with the *E. tirucalli* latex, all three genes were induced ranging from six-fold (BZLF1) to 19-fold (gp350) over background levels demonstrating that *E. tirucalli* latex activates expression of EBV lytic cycle genes.

**Figure 2:**
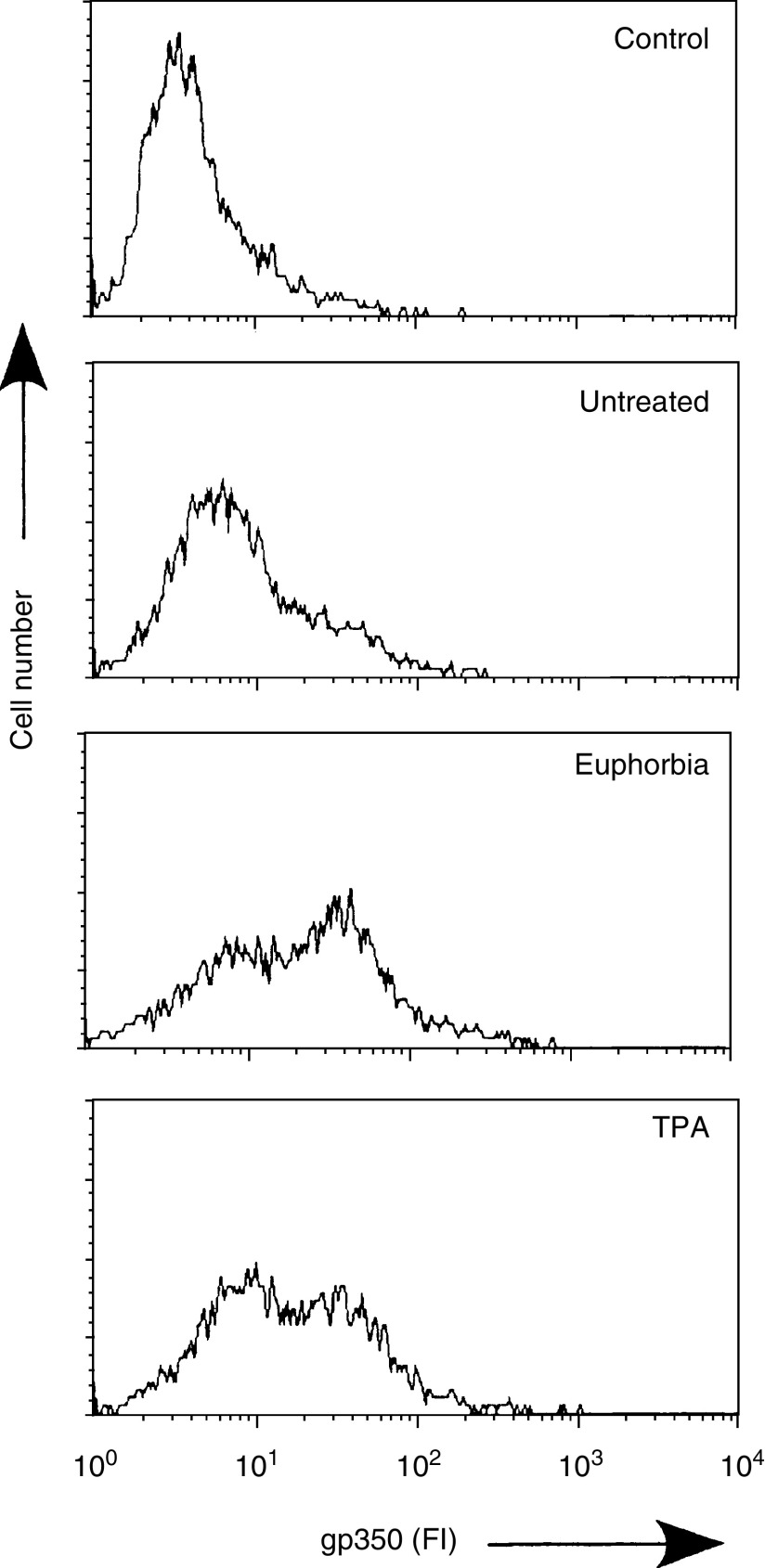
Expression of gp350 in Jijoye cells. Jijoye cells left untreated, treated with 10^−3^ dilution of *E. tirucalli* (Euphorbia) or with 10 ng ml^−1^ TPA (TPA) were examined by IF staining and FCF analysis for levels of gp350 expression. Control indicates IF staining with secondary antibody alone. TPA=12-*O*-tetradecanoyl phorbol-13-acetate; FI=fluorescence intensity.

Flow cytofluorometric analysis was done to determine the percentage of cells expressing gp350 following treatment with the *E. tirucalli* latex as well as to directly compare the efficiency of *E. tirucalli* latex to TPA in the induction of EBV lytic cycle. Jijoye cells were treated with *E. tirucalli* latex diluted to 10^−3^ in CM or with 10 ng ml^−1^ of TPA and incubated for 6 days. A low level of gp350 expression was observed in the untreated cells. Treatment with *E. tirucalli* latex or TPA resulted in increasing number of cells expressing gp350 (Figure 2

). However, we observed a slightly greater induction of EBV gp350 expression in the *E. tirucalli*-treated cells (2.9-fold over background) compared to the cells treated with TPA (2.3-fold).

### Induction of EBV lytic cycle genes by *E. tirucalli* is dose dependent

**Figure 3:**
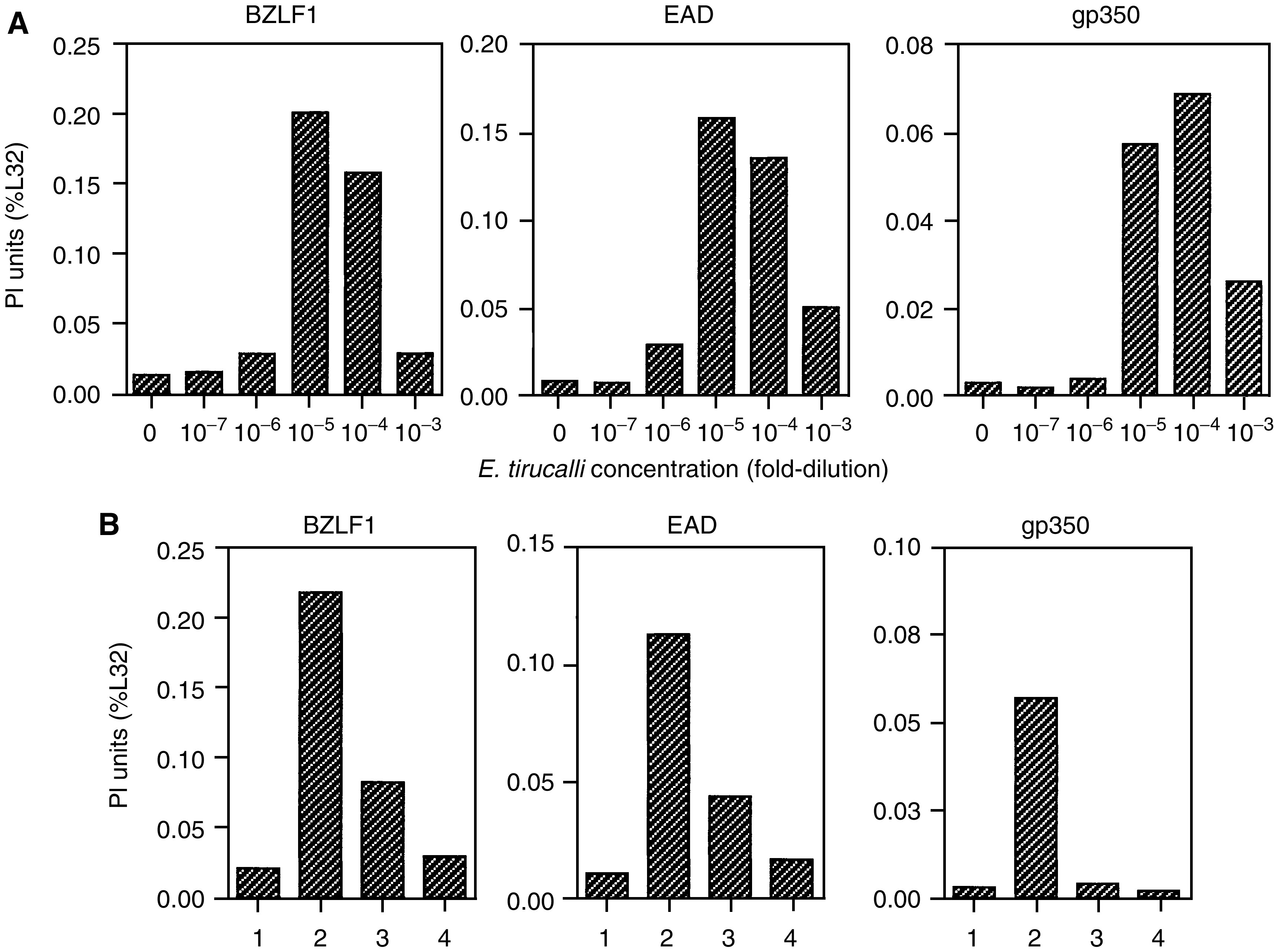
*E. tirucalli* latex activation of EBV lytic gene expression is dose-dependent and PKC-inhibited (**A**) Jijoye cells were treated with 10-fold serial dilutions of *E. tirucalli* latex ranging from 10^−3^ to 10^−7^ and, after 5 days, RNA was extracted and analysed by multiprobe RPA for the measurement of EBV gene expression. The PI counts for each protected probe fragment were obtained, and the data are presented as a percentage of the internal housekeeping (i.e. L32) signal present in each lane. The average values from three experiments are presented. (**B**) Jijoye cells were untreated (1); treated for 5 days with 10^−4^ *E. tirucalli* latex (*E*.*t*.) (2); treated with *E*.*t*. latex and 20 mM PKC-I (3); and 100 mM PKC-I (4), RNA was extracted and analyzed by λ RPA. Quantitation was carried out as described in A. The mean from duplicate samples is shown and is a representative experiment from three separate experiments performed.

To determine if *E. tirucalli* latex had a dose-dependent effect on lytic cycle gene expression, Jijoye cells were treated with 10-fold serial dilutions of latex ranging from 10^−3^ to 10^−7^. Cells were incubated for 5 days and the level of EBV lytic gene activation was assayed for by RPA (Figure 3A

). We observed that at dilutions of 10^−3^ to 10^−6^, lytic gene expression was induced; the highest level of induction occurred at the 10^−4^ and 10^−5^ dilution of the latex. These data demonstrate that even very dilute levels of latex can induce lytic gene expression and suggest that exposure to the latex is biologically relevant for reactivation of EBV lytic cycle genes.

### *E. tirucalli* induces EBV lytic cycle gene transcription by activation of protein kinase C

Induction of the BZLF1 gene by TPA occurs through activation of the protein kinase C (PKC)-dependent pathway (Baumann *et al*, 1998). To determine if *E. tirucalli* latex activation of EBV lytic cycle also occurs through the PKC pathway, Jijoye cells were treated with *E. tirucalli* latex diluted to 10^−3^ in the presence of a PKC inhibitor (PKC-I), 1-(5-isoquinolinesulphonyl)-2-methylpiperazine dihydrochloride. Cells were incubated for 5 days and an RPA was done to measure EBV lytic gene activation. Figure 3B shows the effects of PKC-I on BZLF1, EAD and gp350 gene expression. The activation of *E. tirucalli* latex-induced lytic gene expression was inhibited in the presence of 100 mM PKC-I and, to a lesser extent, 20 mM PKC-I. These data suggest that *E. tirucalli* latex-induced lytic cycle gene expression occurs through the activation of a PKC pathway.

## DISCUSSION

In this study, we found that very dilute concentrations of the unpurified *E. tirucalli* latex were effective at inducing viral reactivation in an EBV^+^ BL cell line, suggesting that direct exposure to the latex could contribute to alterations in the EBV : host balance in humans. Previous studies have examined the carcinogenic effects of methanol extracts from *E. tirucalli* roots, leaves and stems (Mizuno *et al*, 1983; Osato *et al*, 1987), but this is the first study to specifically examine and quantify EBV lytic cycle gene expression in response to direct exposure to the unpurified latex of the *E. tirucalli* plant. Although the milky latex contains a variety of biologic mediators, inhibition of viral reactivation in the presence of a specific inhibitor of PKC suggests that the biologic activity in the latex is the 4-deoxyphorbol ester.

Despite the *in vitro* evidence that indicates the possible role of 4-deoxyphorbol ester from the latex of *E. tirucalli* in the pathogenesis of BL, very little research has looked at how exposures to the latex of this plant occur in human populations. Anecdotal reports suggest that this plant is used as a medication to treat a variety of ailments (Osato *et al*, 1987, 1990). We have conducted preliminary field studies and have observed *E. tirucalli* to be grown abundantly as fencing around homes in the Lake Victoria region of Kenya. We found, in agreement with Osato *et al* (1987), that this plant is used as a herbal medicine but that the different parts of the plant used for medicinal purposes are boiled prior to use, which could potentially inactivate the 4-deoxyphorbol ester. Interestingly, the latex is commonly used as a play item, as glue, and as a topical medicine, exposures we view as being more likely to occur in children. Based on our observations that latex was capable of significantly inducing reactivation of EBV lytic cycle gene expression at dilutions as low as 10^−6^, we hypothesise that children might be inadvertently exposing themselves to the latex by placing their hands in the mouth, a common behaviour in children. The latex would then be absorbed by mucosal surfaces of the mouth and gastrointestinal tract. Mucosal B cells latently infected with EBV could be induced to reactivate the viral lytic cycle. Increased production of infectious virus in the context of malaria-induced immunosuppression would have the potential to result in even greater numbers of EBV-infected B cells. Interestingly, in the prospective study of eBL by de-The *et al* (1978), increases in antibody titres to EBV lytic cycle EA and VCA antigens preceded the emergence of eBL, suggesting that an increase in lytic viral replication occurs prior to the development of eBL. Furthermore, a majority of eBL have cells that express viral lytic genes (Labrecque *et al*, 1999) and have increased titres to BZLF1 antibodies (Ong *et al*, 2001) The identification of *E. tirucalli* latex as a potent activator of viral replication suggests that *E. tirucalli* could act as a critical environmental factor in the genesis of eBL.
